# Immersion challenge model for *Flavobacterium psychrophilum* infection of Atlantic salmon (*Salmo salar* L.) fry

**DOI:** 10.1111/jfd.13699

**Published:** 2022-08-17

**Authors:** Valeria Macchia, Makoto Inami, Anne Ramstad, Fabian Grammes, Andrew Reeve, Thomas Moen, Jacob Seilø Torgersen, Alexandra Adams, Andrew P. Desbois, Rowena Hoare

**Affiliations:** ^1^ Institute of Aquaculture University of Stirling Stirling UK; ^2^ VESO Vikan Namsos Norway; ^3^ AquaGen Trondheim Norway; ^4^ AquaGen Scotland Ltd Stirling University Innovation Park Stirling UK

**Keywords:** Atlantic salmon, bacterial cold‐water disease (BCWD), *Flavobacterium psychrophilum*, rainbow trout fry syndrome (RTFS)

## Abstract

*Flavobacterium psychrophilum* is the causative agent of bacterial cold‐water disease (CWBD) and rainbow trout fry syndrome (RTFS), which affect salmonids. To better understand this pathogen and its interaction with the host during infection, including to support the development of resistant breeds and new vaccines and treatments, there is a pressing need for reliable and reproducible immersion challenge models that more closely mimic natural routes of infection. The aim of this present study was to evaluate a challenge model developed previously for rainbow trout for use in Atlantic salmon. First, preliminary challenges were conducted in Atlantic salmon (*n* = 120) and rainbow trout (*n* = 80) fry using two *F. psychrophilum* isolates collected from each fish species, respectively; fish had been pretreated with 200 mg/L hydrogen peroxide for 1 h. Thereafter, the main challenge was performed for just one *F. psychrophilum* isolate for each species (at 2 × 10^7^ CFU/mL) but using larger cohorts (Atlantic salmon: *n* = 1187; rainbow trout: *n* = 2701). Survival in the main challenge was 81.2% in Atlantic salmon (21 days post‐challenge) and 45.3% in rainbow trout (31 days post‐challenge). Mortalities progressed similarly during the preliminary and main challenges for both species, demonstrating the reproducibility of this model. This is the first immersion challenge model of *F. psychrophilum* to be developed successfully for Atlantic salmon.

## 1 INTRODUCTION

Flavobacteriosis is a bacterial disease caused by the Gram‐negative bacterium *Flavobacterium psychrophilum* that affects freshwater salmonids worldwide (Loch & Faisal, 2018). In rainbow trout (*Oncorhynchus mykiss* Walbaum, 1792), which is particularly susceptible to *F. psychrophilum* infection, this bacterium causes rainbow trout fry syndrome (RTFS) that can result in high mortalities (up to 70%) and even survivors may develop detrimental deformities (Nematollahi et al., 2003). Clinical signs of RTFS include erosion of tissues, particularly of the caudal fin, lower jaw skin ulcerations, pale or necrotic gills, excess mucus production, a pale liver and kidney, enlarged spleen and spinal abnormalities (Barnes & Brown, 2011). Infection with *F. psychrophilum* is also referred to as bacterial cold‐water disease (BCWD) and the bacterium has been associated with occasional cases of fin rot and ulceration in Atlantic salmon (*Salmo salar* L.) in Norway (Nilsen, Johansen, et al., 2011). More recently, *F. psychrophilum* has also been isolated from Atlantic salmon fry (<1 g) following several disease outbreaks in Scotland and this has prompted some unease in the industry (e.g. Anonymous, 2021).

The treatment of choice for flavobacteriosis is antibiotics administered in the feed (Sundell et al., 2019), but there is concern about the development of antibiotic resistance (Ngo et al., 2018). A commercial oil‐based vaccine against *F. psychrophilum* is available to Atlantic salmon farmers in Chile (ALPHA JECT® IPNV‐Flavo), however, this vaccine must be delivered by injection, and this is not suitable for fry which is when the fish are most vulnerable to this pathogen (Wahli & Madsen, 2018). The selection of families of fish with resistance or decreased susceptibility to this pathogen, or genomic selection that exploits genetic markers to calculate the genomic estimated breeding values of selection candidates, offer another possibility for addressing the problems posed by *F. psychrophilum,* and several recent studies have demonstrated the potential of such approaches (Fraslin et al., 2020; Liu et al., 2018; Vallejo et al., 2017; Wiens et al., 2013).

To select for resistant breeds of fish, or to develop new and more effective vaccines to protect against *F. psychrophilum*, including those delivered orally or by immersion (Bøgwald & Dalmo, 2019; Ghosh et al., 2016), reliable and reproducible challenge models are required. Challenge models for *F. psychrophilum* have long been available for rainbow trout (Cipriano & Holt, 2005; Madsen & Dalsgaard, 1999) and, more recently, have been developed for Atlantic salmon (Bruce et al., 2021; Fredriksen et al., 2016). In these models, typically, naïve fry are infected with a known dose of the pathogen by subcutaneous, intraperitoneal or intramuscular injection, with disease progression then assessed by the onset of clinical signs, morbidities and mortalities. Whilst this method of delivery to establish the infection provides standardized and reproducible models with generally high mortality, injection does not well reflect the natural route of infection because it bypasses the primary host defences of the skin and mucus (Koshio, 2016). Establishing infections by injection is also relatively labour intensive, especially when larger groups of fish are included such as in studies for genetic resistance (Plant & LaPatra, 2011).

Challenge by immersion (i.e. bathing the fish in a suspension of *F. psychrophilum*) is closer to the natural route of infection, however, morbidity and mortality rates tend to be more variable, which makes comparisons between experimental groups and studies challenging (Decostere et al., 2000). Indeed, attempts to develop an immersion challenge model for rainbow trout have produced inconsistent results, with some studies documenting only low mortality after challenge (Decostere et al., 2000; Madetoja et al., 2000). Meanwhile, greater mortalities have been achieved by pretreating the fish prior to challenge with hydrogen peroxide to remove the surface mucus (Henriksen et al., 2013; Hoare et al., 2017) or through physically disrupting the fish skin by scarification (Long et al., 2014). Still, no immersion challenge model has been developed successfully for Atlantic salmon.

Hence, the aim of this present study was to develop an immersion challenge model for *F. psychrophilum* in Atlantic salmon fry based on a previous model developed for rainbow trout (Hoare et al., 2017), and to compare rainbow trout and Atlantic salmon challenges for reliability and reproducibility when establishing the infection with different bacterial isolates.

## 2 MATERIALS AND METHODS

### 2.1 Bacterial isolates, culture media and growth conditions

The *F. psychrophilum* isolates used in this present study were recovered from moribund fry at commercial fish farms in Scotland. *F. psychrophilum* 18_S and *F. psychrophilum* 6_S were isolated in 2018 from Atlantic salmon, while *F. psychrophilum* 19_5 and *F. psychrophilum* 356a were isolated in 2019 and 2020, respectively, from rainbow trout. At the farms, spleens were aseptically removed from moribund fish and streaked across plates of modified veggietone agar (MVA), consisting of 5 g/L Veggitones GMO‐free soya peptone (Oxoid, UK), 0.5 g/L yeast extract (Oxoid), 0.5 g/L magnesium sulphate heptahydrate (Fisher Chemicals, UK), 0.2 g/L anhydrous calcium chloride (Thermo Fisher Scientific, USA), 2 g/L dextrose (Oxoid) and 15 g/L bacteriological agar (Oxoid); agar was omitted when the liquid medium (MVB) was required. Inoculated agar plates were incubated at 15°C for 72–96 h. Then, predominant representative colonies were confirmed to be *F. psychrophilum* by a nested PCR that targets the *16S rRNA* gene, as described by Ngo et al. (2017). The nucleotide sequence of *16S rRNA* from each bacterial isolate was >98% identical to the corresponding sequence of *F. psychrophilum* JIP02/86 (also known as ATCC 49511, DSM 21280 or CIP 103535; GenBank accession number AM398681.2). This strain was used for comparison because the complete genome sequence is available (Duchaud et al., 2007) and it has been used similarly in previous studies (Ngo et al., 2017). Each isolate was stored long‐term on Protect™ beads (Technical Services Consultants Ltd., UK) at −70°C. Prior to the challenge, isolates were revived by plating onto MVA and incubating at 15°C for 72–96 h. Then, a single colony of each isolate was inoculated into 3 ml MVB and incubated at 15°C for 72 h at 150 rpm (starter culture). Following this, the starter culture was inoculated into 27 ml MVB, incubated as before and finally introduced into 150 ml MVB at 15°C for 24 h at 150 rpm. Prior to the challenge, the optical density at 525 nm (OD) of the culture was adjusted with phosphate‐buffered saline (PBS, pH 7.3) to 1.0 AU (ca. 2 × 10^8^ colony forming units [CFU]/mL), with an accurate CFU/mL value determined by the 10‐fold plate dilution method of Miles et al. (1938).

### 2.2 Fry

Atlantic salmon (1.8 ± 0.2 g) and rainbow trout (1.9 ± 0.2 g) fry were obtained from AquaGen Norway (ca. 500 and 200 full‐sib families, respectively) and transported to the research facility at Veso Vikan (Namsos, Norway) where the challenge trials were performed. The fish were acclimated for 14 days in glass fibre tanks containing 120 L of well‐aerated flow‐through freshwater. Oxygenation was maintained at >70% in effluent water (0.8 L/kg/min). The water in the tanks was maintained at 12 ± 1°C and a photoperiod of 12:12 light:dark was in operation. The fish were fed a commercial Atlantic salmon feed (Skretting AS, Norway) continuously by an automatic feeder at a rate of 2% bodyweight per day. The *F. psychrophilum*‐free status of the fry was determined by streaking head kidney and spleen samples of ten fish onto MVA and incubating at 15°C for 72–96 h (all were negative for *F. psychrophilum* colonies after this incubation; data not shown).

### 2.3 Immersion challenges

#### 2.3.1 Preliminary challenges

Preliminary challenges were conducted to determine the bacterial isolates and concentration to use for the main challenge. In 10 L tanks containing 8 L of culture water each, four groups of Atlantic salmon fry (*n* = 30 in each tank; ca. 2.4 g) and two groups of rainbow trout (*n* = 40 in each tank; ca. 1.5 g) were starved for 24 h prior to challenge. Each group of fry was immersed in hydrogen peroxide (200 mg/L) for 1 h in aerated tanks containing 4 L of culture water, with water flow stopped. Immediately thereafter, groups of Atlantic salmon fry were placed in replicate tanks containing 4 L of 2 × 10^6^ or 2 × 10^7^ CFU/mL of either bacterial isolate (*F. psychrophilum* 18_S or *F. psychrophilum* 6_S). Meanwhile, groups of rainbow trout fry were placed in replicate tanks containing 4 L of 2 × 10^7^ CFU/mL of *F. psychrophilum* 19_5 or *F. psychrophilum* 356a. The challenges for both fish species were performed for 4 h at 12 ± 1°C, with water flow stopped. After the challenge, the fish were returned to the 10 L tanks, the water flow was returned to normal and the fish were fed as described in Section 2.2. Morbidities and mortalities were recorded each day until the end of the trial (27 days for Atlantic salmon and 19 days for rainbow trout). Moribund fish were euthanised with an overdose of benzocaine chloride (Sykehusapoteket Oslo, Ullevål, Norway) and recorded as dead. Head kidney and spleen were removed aseptically and plated across MVA for 10% of daily mortalities. The agar plates were incubated as Section 2.2 before a representative colony from each plate resembling *F. psychrophilum* was analysed by PCR as described in Section 2.1 to confirm its identity.

#### 2.3.2 Main challenge

The main challenge was performed for Atlantic salmon (*n* = 1187; 1.8 ± 0.2 g) and rainbow trout (*n* = 2701; 1.9 ± 0.2 g) fry that had been starved for 24 h. The fish were placed into two separate aerated tanks containing 90 L of hydrogen peroxide (200 mg/L) for 1 h. Then, the fish were returned to the 120 L glass fibre acclimation tanks. Water flow was stopped in each tank and the water volume reduced to 54 L. For each challenge, 6 L of bacteria was added to the tank water to give a final concentration of 2 × 10^7^ CFU/mL. Atlantic salmon were challenged with *F. psychrophilum* 6_S while rainbow trout were challenged with *F. psychrophilum* 19_5. After 4 h, the water flow recommenced, the tank water volume returned to 120 L and the fish were fed as described in Section 2.2. Morbidities and mortalities were recorded each day until the end of the trial (21 days post‐challenge [dpc] for Atlantic salmon and 31 dpc for rainbow trout), and samples were taken for bacteriology according to Section 2.3.1.

### 2.4 Statistical analysis

Kaplan–Meier survival curves were generated using Excel and log‐rank tests were performed using R package *survminer* (Version 0.4.9; Kassambara et al., 2017) and *xfun* (Version 0.30; Xie et al., 2018). Log‐rank tests were used to compare differences in percentage survival for each fish species for the different isolates and bacterial challenge concentrations. This statistical test was also used to compare the preliminary and main challenges for each fish species at the same bacterial concentration. *p*‐values <.05 were considered to indicate a statistically significant difference.

### 2.5 Ethics statement

All experimental procedures with live fish were carried out in accordance with the Norwegian Food Safety Authority guidelines and were approved by the Animal Welfare and Ethical Review Body of the University of Stirling, UK.

## 3 RESULTS

### 3.1 Preliminary challenges

**FIGURE 1:**
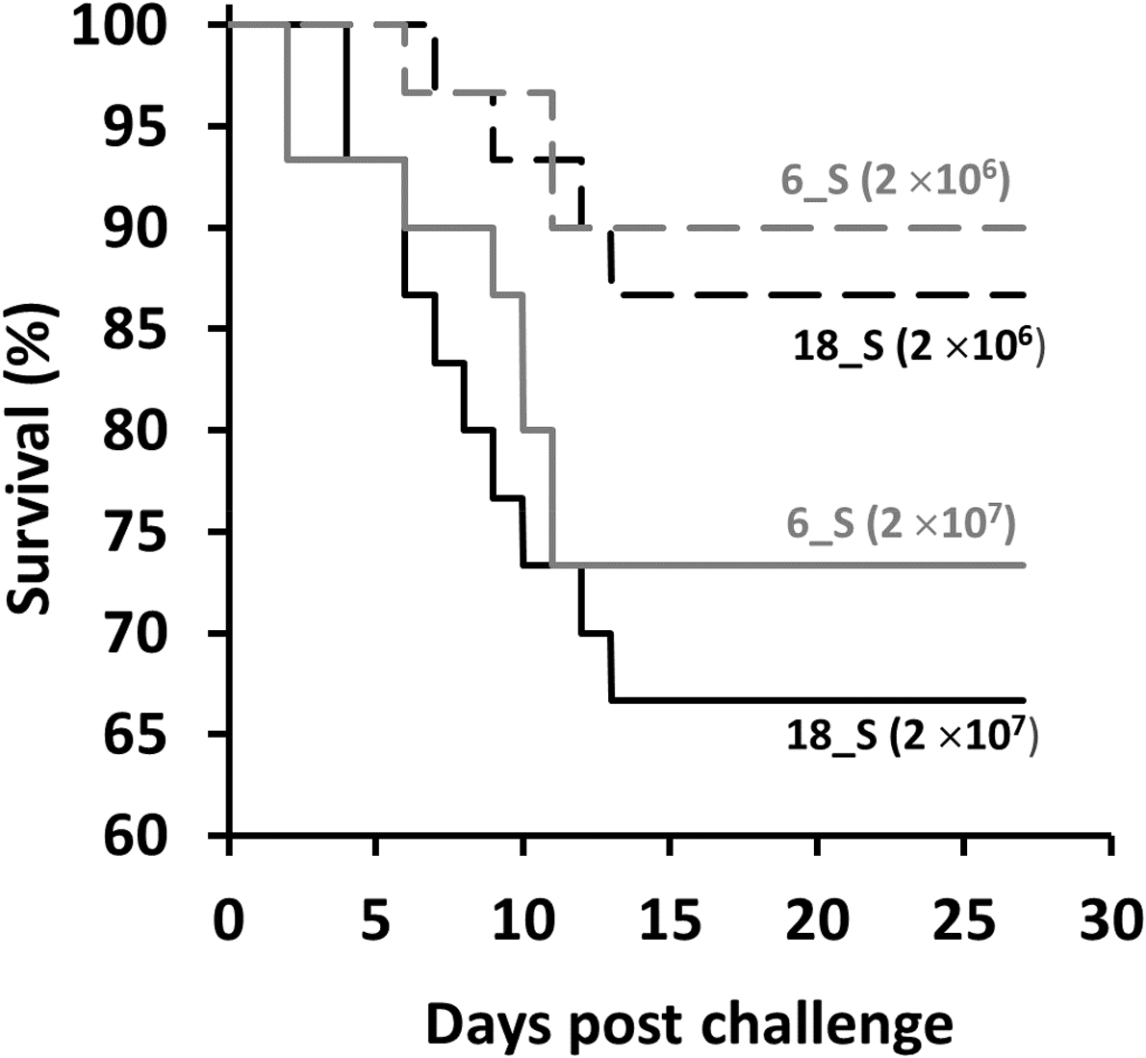
Kaplan–Meier survival curves of Atlantic salmon in the preliminary challenge with two isolates derived from Atlantic salmon (*Flavobacterium psychrophilum* 6_S and *F. psychrophilum* 18_S) at two concentrations, specifically 2 × 10^6^ and 2 × 10^7^ CFU/mL. Fish with a mean mass of 2.4 ± 0.2 g (*n* = 30 per treatment group) were exposed to hydrogen peroxide (200 mg/L) for 1 h prior to immersion challenge with the bacteria for 4 h

The preliminary challenge of Atlantic salmon fry showed that both *F. psychrophilum* 18_S and *F. psychrophilum* 6_S were virulent in a dose‐dependent manner, with first mortalities observed at 2 dpc for *F. psychrophilum* 6_S at 2 × 10^7^ CFU/mL and 4 dpc for *F. psychrophilum* 18_S at 2 × 10^7^ CFU/mL; no mortalities occurred in any group after 13 dpc (Figure 1). *F. psychrophilum* 6_S was significantly more virulent at the higher concentration (χ^2^ = 5.77 dof = 1, p < 0.05) and resulted in 73.3% survival at 27 dpc compared with 90% survival in the 2 × 10^6^ CFU/mL group. By contrast, for *F. psychrophilum* 18_S survival reached 66.7% in the 2 × 10^7^ CFU/mL group compared with 85.7% in the 2 × 10^6^ CFU/mL group, and the difference between the survival in these infected groups failed to reach statistical significance (χ^2^ = 3.03, dof = 1, *p* = .08). There were no significant differences in survival between the bacterial isolates at either of the concentrations used to challenge the Atlantic salmon fry (χ^2^ = 6.10, dof = 3, *p* > .05). Colonies suspected to be *F. psychrophilum* morphologically were recovered from plating Atlantic salmon tissues across MVA for 71.6% to 100% of the fish sampled (Table 1). A representative colony suspected to be *F. psychrophilum* from each culture plate was confirmed to be *F. psychrophilum* by PCR, with all the colonies examined testing positive by this analysis.

**TABLE 1:** Outline of the preliminary and main challenges with *Flavobacterium psychrophilum* in Atlantic salmon and rainbow trout, including the fish culture conditions, bacterial isolates used, the challenge concentrations, and percentage of sampled mortalities where *F*. *psychrophilum* was recovered by bacteriological culture and confirmed by PCR.

	Tank number (volume; number of fish; mean fish mass)	Bacterial isolate	Fish species	Bacterial challenge concentration (CFU/ml)	Duration of trial (days post‐challenge)	Survival at end of trial (%)	Proportion of fish tested that yielded *F*. *psychrophilum* colonies (%)
Preliminary challenges	1 (10 L; *n* = 30; 2.4 g)	*F. psychrophilum* 6_S	Atlantic salmon	2 × 10^6^	27	90.0	72.0
	2 (10 L; *n* = 30; 2.4 g)	*F. psychrophilum* 6_S	Atlantic salmon	2 × 10^7^	27	73.3	100.0
	3 (10 L; *n* = 30; 2.4 g)	*F. psychrophilum* 18_S	Atlantic salmon	2 × 10^6^	27	85.7	71.6
	4 (10 L; *n* = 30; 2.4 g)	*F. psychrophilum* 18_S	Atlantic salmon	2 × 10^7^	27	66.7	79.0
	5 (10 L; *n* = 40; 1.5 g)	*F. psychrophilum* 19_5	Rainbow trout	2 × 10^7^	19	32.5	100.0
	6 (10 L; *n* = 40; 1.5 g)	*F. psychrophilum* 356a	Rainbow trout	2 × 10^7^	19	95.0	100.0
Main challenge	1 (120 L; *n* = 1,187; 1.8 g)	*F. psychrophilum* 6_S	Atlantic salmon	2 × 10^7^	21	81.2	79.0
	2 (120 L; *n* = 2,701; 1.9 g)	*F. psychrophilum* 19_5	Rainbow trout	2 × 10^7^	31	45.3	85.7

**FIGURE 2:**
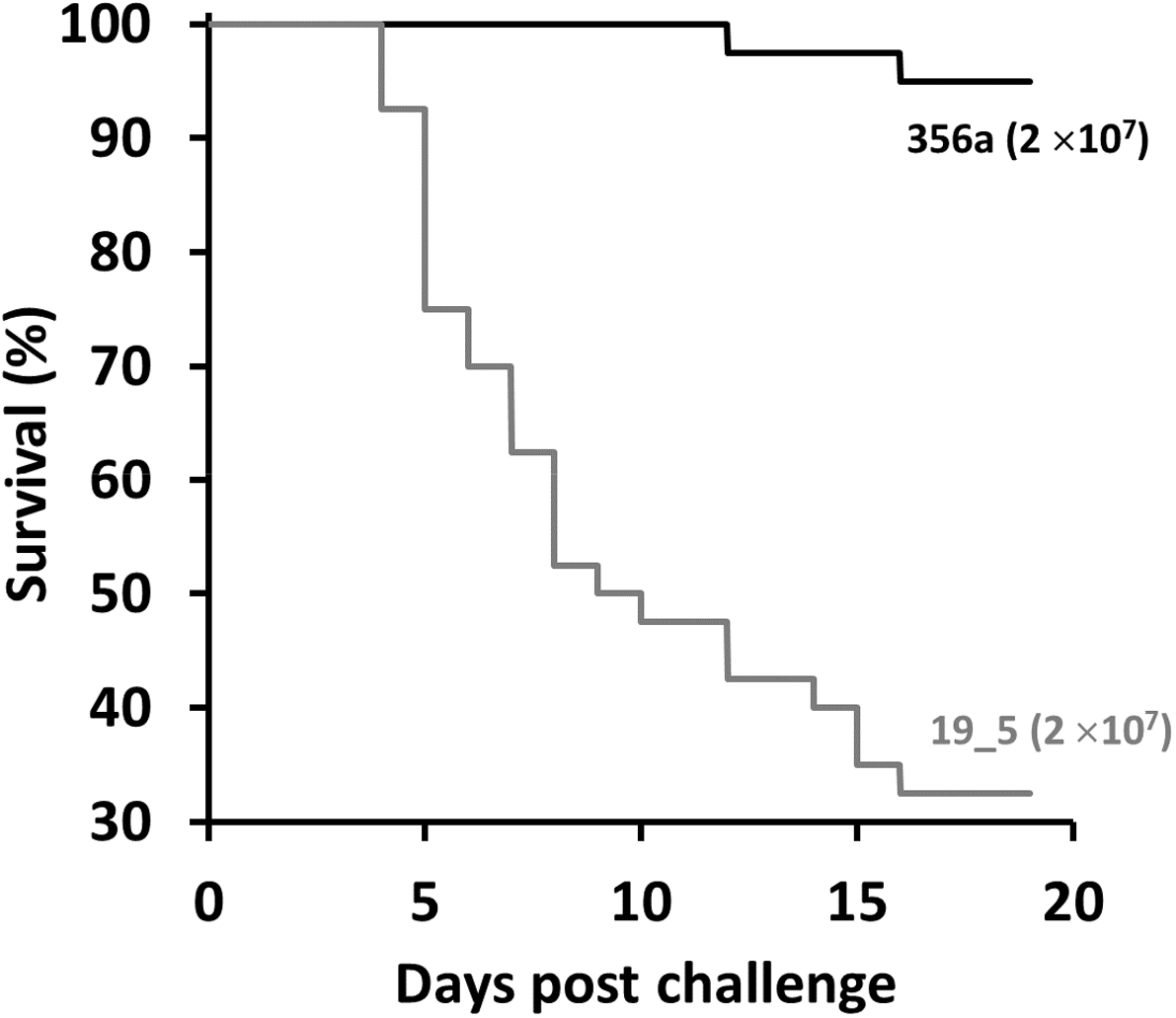
Kaplan–Meier survival curves of rainbow trout in the preliminary challenge with two isolates derived from rainbow trout (*Flavobacterium psychrophilum* 356a and *F. psychrophilum* 19_5) at 2 × 10^7^ CFU/mL. Fish (*n* = 80) with a mean mass of 1.5 ± 0.2 g (*n* = 40 per treatment group) were exposed to hydrogen peroxide (200 mg/L) for 1 h prior to immersion challenge with the bacteria for 4 h

Meanwhile, the preliminary challenge in rainbow trout showed that *F. psychrophilum* 19_5 at 2 × 10^7^ CFU/mL was highly virulent and survival was 32.5% at 19 dpc. By contrast, *F. psychrophilum* 356a at 2 × 10^7^ CFU/mL resulted in 95% survival by the end of the trial (Figure 2). There was a statistically significant difference in rainbow trout fry survival between the two *F. psychrophilum* isolates used (χ^2^ = 14.8, dof = 1, *p* < .05). Each fish sampled by bacteriology yielded colonies on MVA that were suspected to be *F. psychrophilum* (Table 1) and this identity was confirmed for a representative colony from each culture plate by PCR.

### 3.2 Main challenge

**FIGURE 3:**
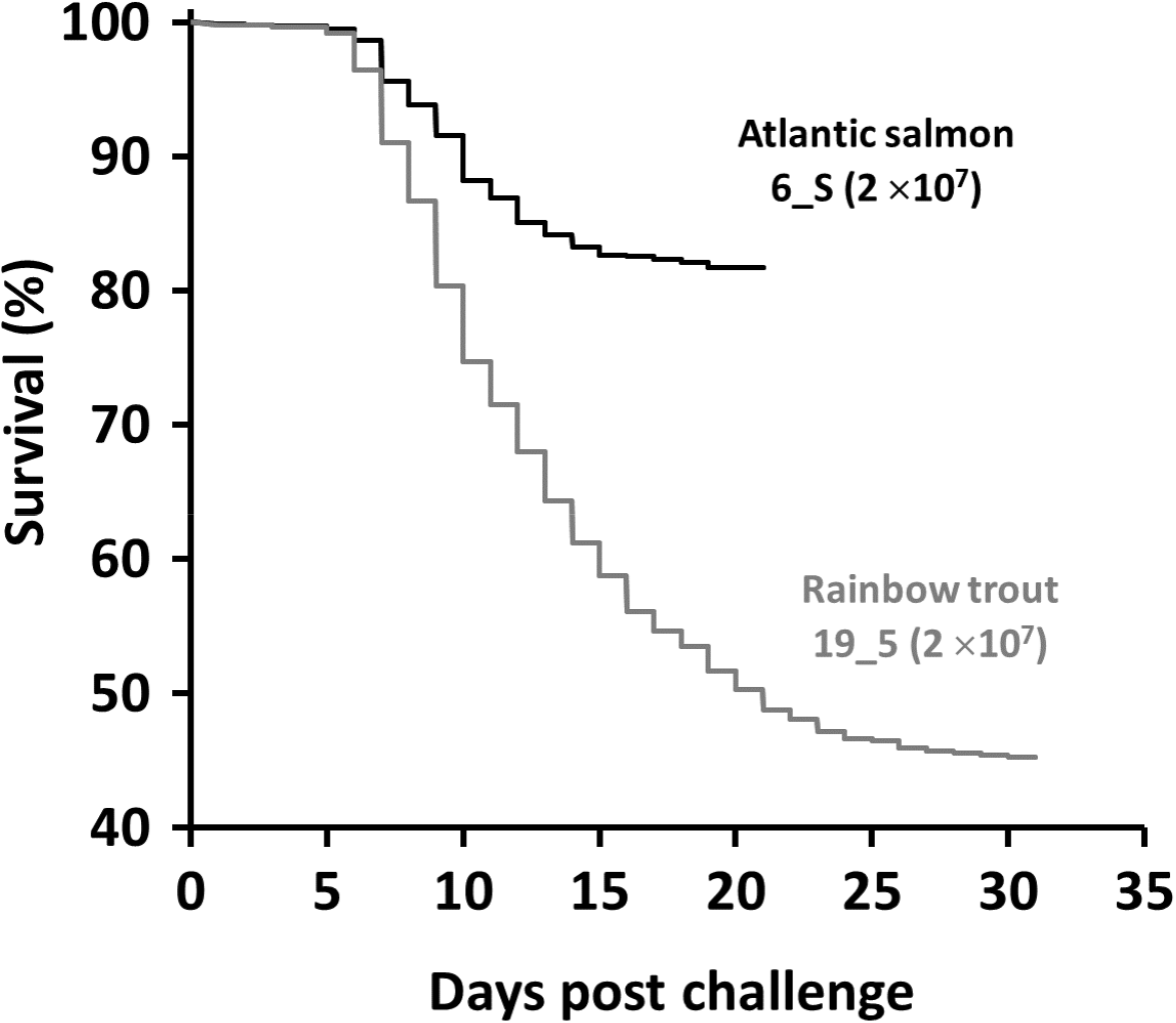
Kaplan–Meier survival curves of Atlantic salmon and rainbow trout in the main challenge with *Flavobacterium psychrophilum* 6_S (for Atlantic salmon) and *F. psychrophilum* 19_5 (for rainbow trout) at 2 × 10^7^ CFU/mL. Atlantic salmon (*n* = 1187; 1.8 ± 0.2 g) and rainbow trout (*n* = 2701; 1.9 ± 0.2 g) were exposed to hydrogen peroxide (200 mg/L) for 1 h prior to immersion challenge with the bacteria for 4 h

The main challenge was carried out using 2 × 10^7^ CFU/mL of *F. psychrophilum* 6_S and *F. psychrophilum* 19_5 for the Atlantic salmon and rainbow trout challenges, respectively. The survival for Atlantic salmon and rainbow trout at the end of the main challenges was 81.2% (at 21 dpc) and 45.3% (at 31 dpc), respectively (Figure 3). Similar to the preliminary challenges, no external lesions were observed on dead or moribund fish. Colonies suspected to be *F. psychrophilum* were detected by culture for 79.0% and 85.7% of Atlantic salmon and rainbow trout tissues plated across MVA, respectively. PCR of a representative colony from each culture plate was performed and those tested were positive by this analysis in each case. There were no significant differences in percentage survival of Atlantic salmon and rainbow trout between the preliminary and main challenges for *F. psychrophilum* 6_S (χ^2^ = 2, dof = 1, *p*= > .05) and *F. psychrophilum* 19_5 (χ^2^ = 0.1, dof = 1, *p* > .05) performed at the same doses, respectively (Table 1).

## 4 DISCUSSION

Flavobacteriosis caused by the Gram‐negative, psychrophilic bacterium *F. psychrophilum* affects rainbow trout and Atlantic salmon, especially at the fry stage when the fish are too small to be vaccinated by injection. To address the need for a reliable challenge model that can underpin future disease mitigation efforts, an immersion challenge model for *F. psychrophilum* in Atlantic salmon fry was developed for the first time. This new challenge model was based on a previous model that had been used for *F. psychrophilum* in rainbow trout (Hoare et al., 2017). A rainbow trout challenge was conducted as a point of comparison to evaluate reliability and reproducibility when establishing the infection with different isolates of the bacterium. The immersion challenge models were reproducible, as evidenced by the non‐significant differences in percentage survival between the preliminary and the main challenges for both Atlantic salmon and rainbow trout when the same bacterial concentrations and isolates were used.

Previous experimental research on *F. psychrophilum* infection of salmonids, including Atlantic salmon and rainbow trout, has relied on inducing mortality via intramuscular injection of the bacteria (Fredriksen et al., 2016). However, injection delivery bypasses the physical and immune barriers of the skin and the mucosa, which play an important role in protecting the fish against infection (Koshio, 2016). Long et al. (2014) performed an immersion challenge and observed that mortality was significantly greater in fish that had been scarred compared to those that had not been pretreated in this way (81.5 vs. 19.4%). Similar mortality rates have been reported where fry were compromised with an incision below the dorsal fin prior to challenge by immersion (Madetoja et al., 2000). These findings suggest that disruption of the skin surface facilitates the entering of the host and the subsequent establishment of the infection for *F. psychrophilum*. Moreover, Hoare et al. (2022) highlighted the importance of rainbow trout mucus during immersion challenge studies with *F. psychrophilum*. In particular, greater levels of actins were observed in the skin mucus of rainbow trout fry challenged by immersion compared with injection delivery, and the authors suggest these may have a role in mucosal immunity by potentiating the macrophage response to pathogens (Hoare et al., 2022). In this present study, the fish were pre‐treated with hydrogen peroxide prior to bacterial challenge to remove some of the surface mucus and expose the skin to the bacteria. This approach has been used previously to establish the effective immersion challenge model for *F. psychrophilum* in rainbow trout (Henriksen et al., 2013; Hoare et al., 2017). In addition to serving as a barrier, rainbow trout and Atlantic salmon have bactericidal enzymes and other antimicrobial compounds in the surface mucus (Ángeles Esteban, 2012; Kelly & Salinas, 2017; Sprague & Desbois, 2021). Furthermore, Fast et al. (2002) observed that the Atlantic salmon mucus exhibits protease and lysozyme activities and abundant mucous cells are found in the epidermis. Hence, when designing this challenge model for Atlantic salmon, the pre‐treatment step was used to strip the mucus prior to the challenge with a large dose of *F. psychrophilum* (2 × 10^7^ CFU/mL) in an attempt to ensure that an infection would be established.

Despite the lack of external lesions in moribund or deceased fish, re‐isolation rates of *F. psychrophilum* were relatively high (e.g. 85.7% from rainbow trout and 79.0% from Atlantic salmon in the main challenge) and this suggests that the infection was systemic in nature. This result is in line with reports of natural outbreaks, where Nilsen, Olsen et al. (2011) and Rimstad (2019) observed systemic infection with *F. psychrophilum* in rainbow trout hatcheries. *F. psychrophilum* can be difficult to isolate from fish tissues and this may explain why the bacterium was not recovered by culture from each fish sampled (Cepeda et al., 2004).

This present study confirmed little difference in survival between the preliminary and main challenges in both rainbow trout and Atlantic salmon challenges when similar concentrations of the same bacterial isolate were used, which supports the reliability and reproducibility of this model. Of course, further studies involving more tanks and replication, including mock‐challenged control groups, are needed to be completely satisfied with the reproducibility of this model, but the present observations are promising. Notably, biomass density can influence pathogen transmission, host stress levels and mortality rates (Williams et al., 2002; Tort 2011), but the lack of significant differences between the preliminary and main challenges for both species suggests the biomass density differences between the trials had little or no effect on overall mortality. This observation is consistent with Klung et al., (2021) who observed stocking density had no significant effect on the stress response and disease susceptibility of juvenile rainbow trout when infected with infectious haematopoietic necrosis virus. Furthermore, there remains scope to improve this challenge model in Atlantic salmon, for example by determining a lower threshold for the bacterial dose that would still establish an infection reliably, particularly if this led to the development of more classical external clinical signs indicative of surface infection. Additionally, with respect to lethal infection in Atlantic salmon fry, survival was relatively high and it would be highly desirable to attempt to reduce this further for some applications, including vaccine efficacy assessments where >60% mortality is recommended (Amend, 1981).

One logistical issue with carrying out immersion challenges with *F. psychrophilum* is the large quantity of bacteria that is needed and consequent difficulties with washing and preparing the bacteria, including preventing contamination and maintaining high viability (Lagier et al., 2015). In this present study, washing steps for the bacteria were not performed meaning extracellular products were also present for the challenges, and there is some evidence that these may enhance the efficacy of an immersion challenge (Lapatra et al., 2010), though this requires further study to confirm.

In the preliminary challenge for rainbow trout, only *F. psychrophilum* 19_5 was virulent, while *F. psychrophilum* 356a caused <5% cumulative mortalities by the end of the trial. Virulence in *F. psychrophilum* is poorly understood, and differences in virulence of these bacterial isolates are possibly due to the genotypes of the isolates used. Conducting tests with isolates of different genotypes would help to determine the extent of the *F. psychrophilum* isolates for which this newly developed model is useful, whilst providing a platform for understanding differential virulence of strains. Finally, tails were collected from every fish, which will facilitate future genome‐wide association studies to identify determinants of susceptibility to this pathogen.

In conclusion, this is the first study to develop a reliable and reproducible immersion challenge model for *F. psychrophilum* in Atlantic salmon fry. This challenge model, which was confirmed to be effective for rainbow trout too, provides the means to investigate host‐pathogen interactions and allows for the evaluation of new therapies and preventative measures such as resistant fish breeds and vaccines.

## FUNDING INFORMATION

This study was funded by the Sustainable Aquaculture Innovation Centre (SAIC), AquaGen Scotland Ltd and the University of Stirling.

## CONFLICT OF INTEREST

The authors are unaware of any significant conflicts of interest that have influenced this study.

## Data Availability

The data that support the findings of this study are available from the corresponding author upon reasonable request.
